# In Psycho-Spiritual Integrative Therapy for Women with Primary Breast Cancer, What Factors Account for the Benefits? Insights from a Multiple Case Analysis

**DOI:** 10.3390/healthcare3020263

**Published:** 2015-05-12

**Authors:** John Rettger, Kathleen Wall, Diana Corwin, Alexandra N. Davidson, David Lukoff, Cheryl Koopman

**Affiliations:** 1 Department of Psychiatry and Behavioral Sciences, Stanford University, Stanford, CA 94305, USA; E-Mail: jrettger@stanford.edu; 2 Sofia University, 1069 E Meadow Circle, Palo Alto, CA 94303, USA; E-Mails: kewall@sbcglobal.net (K.W.); david.lukoff@sofia.edu (D.L.); 3 Independent Practice, 1069 E Meadow Circle, Palo Alto, CA 94303, USA; E-Mail: dcorwin@paloaltou.edu; 4 Kaiser Permanente, 820 Las Gallinas Avenue, San Rafael, CA 94903, USA; E-Mail: alexandra.n.davidson@kp.org

**Keywords:** integrative cancer care, breast cancer, spirituality, quality of life, group intervention, mindfulness, spiritual well-being

## Abstract

This study sought to understand the context in which Psycho-Spiritual Integrative Therapy (PSIT), a group intervention, promotes varying degrees of spiritual growth and quality of life change in breast cancer survivors. A secondary aim was to explore the relationship between spiritual well-being (SWB) and Quality of Life (QL) in PSIT participants. A qualitative, multiple case analysis was undertaken to examine the experiences of two participants with the highest change scores on the Functional Assessment of Chronic Illness Therapy Spiritual Well-Being Scale-Expanded Version (FACIT-Sp-Ex) and two participants with among the lowest change scores on this measure. The participant factors thought to contribute to SWB and QL changes included utilization of metacognitive psychological skills and spiritual/religious frameworks, while PSIT factors included application of PSIT core intervention components, cognitive restructuring, group dynamics, and the role of the facilitator. The nature and extent of participant use of spiritual practices appeared to shape the relationship between SWB and OL. The findings suggest directions for future research to investigate potential moderators and mediators of treatment efficacy of PSIT specifically, as well as other psycho-spiritual interventions for cancer survivors more generally.

## 1. Introduction

### 1.1. Background Literature

There are approximately 3.1 million women living with breast cancer in the United States today and more than 250,000 more women will be diagnosed in the next year [1]. The prognosis and indicated treatments for breast cancer are impacted by a variety of factors, including the stage of the tumor at diagnosis, the biological characteristics of the tumor, and the health and age of the patient [1]. Several studies have found that the prevalence of mood disorders and psychological distress is higher among cancer patients than in the general population [2,3]. Individuals with a cancer diagnosis may experience heightened vulnerability to stress, anxiety, and depression as they grapple with the possibility of dying from their illness [4,5]. Psychological and physical distress negatively impact quality of life (QL) and can persist as long as years after the termination of treatment [6,7,8].

A growing body of literature demonstrates that Complementary and Alternative Medicine (CAM) interventions can lessen the physical, psychological, and spiritual challenges associated with breast cancer, and cancer patients and survivors are increasingly seeking out CAM interventions to fulfill their spiritual needs [9,10,11]. Many qualitative and quantitative studies have found positive relationships between religion, spirituality, and a sense of well-being [6,12,13,14]. Higher levels of spirituality and faith are associated with higher levels of perceived cancer-related growth in cancer patients, and most cancer patients report a reliance on religious and/or spiritual beliefs to cope with cancer [14,15].

As more evidence establishes the effectiveness of CAM interventions and the popularity of these treatments increases, questions regarding the treatment mechanics and mediating factors continue to arise. Prior research on this topic has been mostly correlational and has not elucidated the experiences of the individual participants [7,8]. While these correlational studies have demonstrated the importance of spirituality in psychological treatment outcomes, a deeper understanding is still needed of the participants’ experiences and personal perspectives of spirituality. Developing such understanding has the potential to improve the translation of these findings into practice recommendations and to develop more effective interventions. This study sought to address this gap in the literature by examining the lived experiences of participants in one type of CAM intervention, Psycho-Spiritual Integrative Therapy (PSIT).

### 1.2. Psycho-Spiritual Integrative Therapy (PSIT)

PSIT is an integrated psychotherapeutic approach explicitly targeting treatment of the whole person. CAM approaches described in the literature for breast cancer patients typically address the dimensions of thought, body, and emotions; most do not specifically aim to integrate spirituality, with few exceptions [5,8,16,17,18]. PSIT specifically addresses physical, psychological, existential, and spiritual factors [19]. Preliminary studies of PSIT have shown promise in promoting health and well-being among breast cancer survivors [20].

PSIT is both an approach to psychotherapy and a spiritual practice focused on a lived spirituality [19,21,22]. As such, PSIT supports breast cancer patients seeking meaning and purpose as well as deepened spirituality. PSIT integrates psychotherapy and spiritual practices in order to help clarify and fulfill the individual’s life purpose and to build skills for resolving obstacles in life transitions, including cancer survivorship. PSIT is non-sectarian, relying on a personal sense of the sacred, and is therefore potentially acceptable to people of most religious/spiritual orientations as well as to people who do not identify as religious/spiritual but who embrace the pursuit of inspiration and life meaning in coping with cancer and other life challenges.

Participants begin the PSIT process by identifying their highest life purpose or aspiration. They then identify thoughts, behaviors, or other factors that help or hinder the attainment of their aspiration. Through in-session activities and independent mindfulness practice, participants are instructed in cultivating the skill of non-judgmental observation. With the aim of non-judgmentally witnessing the aspects that help and hinder attainment of their identified life purpose or aspiration, participants are encouraged to attend to their responses, including bodily sensations, emotions, thoughts, images, and motivations. PSIT aims to help participants to deepen and integrate their awareness of these experiences into their self-understanding by writing and drawing about these experiences in journals and sharing their observations with the group.

Additionally, participants are encouraged to cultivate awareness of their spirituality and how this may assist in fulfilling their aspiration by using passage meditation. Participants share a passage from a prayer, spiritual text, poem, or lyric that for them evokes a personal sense of the sacred (*i.e*., profoundly meaningful and/or inspiring). The passage is repeated slowly to allow the participant to enter into a meditative state. In this meditative state, participants are guided in the surrender of their strivings and refocus their attention on experiencing the sacred—however this is personally conceptualized. This active coping process of surrender is intended to bolster awareness and acceptance of the elements of the self that help and hinder one’s goals [23].

These steps are repeated in every session and in home practice and documented through journal writing. At the conclusion of the intervention, participants reflect on how they have changed during the PSIT process and set goals for fulfilling their aspirational life purpose; they share these insights with the group and receive feedback and support from other members.

The PSIT model has just been described as it has been designed. Previous research has examined aspects of this model among participants in the aggregate, using both quantitative and qualitative methods [20,23,24]. Here, however, we use a different methodological approach, that of multiple case analysis, which examines select participants in depth to explore whether new insights can be gleaned by examining in depth the lived experience of four PSIT participants who participated in PSIT.

### 1.3. Study Focus

This study sought to add to the body of qualitative research describing the relationship between spirituality, SWB, and QL. A multiple case analysis design was chosen to allow deeper inquiry into how and why changes in SWB and QL occur during the PSIT group intervention. Four participants’ experiences were investigated in detail to understand how spirituality is or is not promoted and to further explore the relationship between spirituality and QL. Through this qualitative analysis, we sought to identify both individual participant and PSIT intervention factors that contributed to positive or negative changes in SWB and QL.

Describing individual factors associated with greater or less benefit may identify potential moderators of treatment efficacy for future research on PSIT. Similarly, describing PSIT components associated with greater or less benefit may identify potential mediators of treatment efficacy for future research. Exploring the relationship between spirituality and QL in this context may generate insights about how PSIT can enhance QL through its focus on spirituality.

## 2. Methods

### 2.1. Overview

This study used archival data collected during a larger research study approved by the Institutional Review Board. The intervention was delivered to 2 groups initially comprised of a total of 30 women who met study criteria. PSIT group participants received 24 hours of PSIT intervention delivered across 8 weekly three-hour sessions. Twenty-four participants completed the PSIT group. Two post-treatment follow-up assessments were conducted, one in the week following treatment (*n* = 23) and another conducted in the week following a 3 hour booster session (*n* = 20) conducted one month after the 8th session.

In applying the multiple case analytic approach to this study’s aims, we examined two sets of participants in depth—two of the PSIT participants who reported the greatest improvement in spiritual well-being and two of those who experienced the least improvement over the course of the PSIT group. A multiple case analysis study design was selected to allow the investigation of “how and why” research questions and to examine specific cases within a complex context [25,26].

### 2.2. Measures

Functional Assessment of Cancer Therapy-Breast (FACT-B). The FACT-B uses a 37-item self-report scale to measure multidimensional areas of quality of life for breast cancer patients [27]. Participants rate the veracity of the statements using a Likert scale. It includes questions about physical well-being (e.g., “I have a lack of energy”), social/family well-being (e.g., “I feel close to my friends”), emotional well-being (e.g., “I feel sad), and functional well-being (e.g., “I am able to enjoy life”). Pre and post quality of life scores for all participants were assessed using this scale.

Functional Assessment of Chronic Illness Therapy-Spiritual (FACIT-Sp-Ex). The spiritual well-being of the participants was assessed through the Functional Assessment of Chronic Illness Therapy-Spiritual (FACIT-Sp-Ex) [28]. The FACIT-Sp-Ex is a 23-item instrument that yields scores on two subscales, Meaning/Peace and Spirituality, as well as a total score of Spiritual Well-Being (with a possible range of scores of 0–92). Higher scores on these subscales indicate greater meaning/peace and spirituality, with possible score ranges of 0–32 and 0–16, respectively. Sample questions include “I have a sense of purpose in my life” and “I find comfort in my faith or spiritual beliefs.” For this study, improvement was operationalized by calculating change scores for spiritual well-being (SWB) from the baseline and post-treatment scores obtained with this scale.

### 2.3. Participants

Participants in this study were required to be female, 18 years of age or older, fluent in English, and diagnosed with breast cancer in stages 0–3 within the past 10 years that was non-metastatic at the time of recruitment. Women with psychotic disorders, intrusive suicidal thoughts, or alcohol or drug abuse within the last year were excluded. For this study, participation also required that women provide informed consent and be available to be interviewed. The four women included in this study were married/partnered. Their ages ranged from the low 40s to the low 60s. Three were Caucasian and one was Southeast Asian. At baseline, their breast cancer diagnoses ranged from stage 0–3.

For ease of explanation, we will refer to the four women chosen to participate in this multiple case analysis as “low change” (LC) and “high change” (HC). The 2 LC participants (referred to here with the pseudonyms Jan and Beth) were those with 8% and 11% change scores, including a participant whose change score showed a slight decrease rather than increase from the baseline to post-treatment assessment. Five other participants from the original study showed very small change scores (1%–6%), but were unable to be interviewed for the current study. The 2 HC participants (referred to here with the pseudonyms Carol and Dee) had 53% and 50% change scores on the FACIT-Sp-Ex, demonstrating large improvements. Another participant in the larger study showed a change score of 51% but was not able to be interviewed for this study.

### 2.4. Procedure

Researchers reviewed multiple sources of data from each participant which included semi-structured interviews with participants and journals kept by participants during the larger omnibus study, both sources which will be further described below. Data also included quantitative assessments from the omnibus study [21,26,29]. Triangulation across data sources and cases was used to promote internal and external validity of findings [26,29]. For example, researchers matched interview data with quantitative measurements to examine consistency (e.g., matching a participant’s report of increased SWB with her score on the FACIT-Sp-Ex). Additionally, data from each of these four cases was compared against each other case examined by researchers.

### 2.5. Semi-Structured Interview

The first author of this study conducted 1 to 2 hour semi-structured interviews with each participant. Audio-recorded interviews were transcribed for detailed review by researchers. Interview questions explored the participants’ perceptions of the impact of the PSIT intervention on their spiritual lives. In particular, interview questions sought to elucidate participants’ views of their spiritual growth and changes (from before to following the PSIT intervention) and how these changes may have been related to the participants’ spiritual and religious history.

### 2.6. PSIT Participant Journals

As part of the larger study, PSIT participants used journals to record their experiences and reactions to exercises. Participants brought these journals to their semi-structured interview where the PSIT protocol was used to explore each participant’s creatively expressed work product. The focus of this interview was directed on the participants’ experiences of their work in mind, body, feelings, will, and spirit. If a participant did not bring her workbook to the interview, the interviewer asked permission to contact the participant with any follow-up questions that might arise from workbook review. To archive data, workbooks were digitally copied and stored on a password-protected computer.

### 2.7. Design

The researchers utilized Stake’s procedure for analyzing multiple case studies [29]. Stake’s method includes a number of specific steps, beginning with elaborate note taking to determine how each research question (henceforth referred to as “themes”) is addressed within each particular case [29]. This process facilitates the development of a case formulation incorporating context, observations, and important data sources for each case. Using this method, study data was organized to create a meaningful narrative about each participant’s experience of spirituality while in the PSIT process group. After these case formulations were complete, researchers used a multiple case study analysis procedure to compare and contrast information across cases to develop theme-related findings.

## 3. Results

Below, the spirituality, psychological functioning, and theme-related findings of each individual participant are highlighted.

### 3.1. Jan (LC)

#### 3.1.1. Spirituality

Jan (LC) had a FACIT-Sp-EX pre score of 80 and a post score of 74, which was a 7.5% decrease in SWB. Her FACT-B pre score was 110 and her post score was 107, a 3% decline in QL. Jan self-reported that she was a practicing Catholic. Jan reported that during difficult times she would pray “extra hard” and rely on God for support and assistance. She described using rituals, such as the Catholic rosary, and petitionary prayer in her religious practice. Despite her positive appraisals of her relationship with God, she wrestled spiritually with her breast cancer experience, “When you have this diagnosis, you start questioning why me? And what will my future be?” She described experiencing doubts about whether God was listening to her prayers, “with this experience that [I’ve] been through with cancer, [I] kind of question, does God hear me?” During post-study interviews, Jan reported experiencing gratitude towards God for restoring her health, “I am here, I am healthy, I am grateful for that.” In addition to her spirituality, Jan stated that other factors that gave her strength during her illness were family and cultivating feelings of love and peace.

#### 3.1.2. Psychological Functioning

Jan (LC) described her hinderer to her aspirational life purpose as worry, which was a source of concern for her. Her view of worry was it does not solve anything and it would benefit her to “find a new word for it”. She stated that “it’s not good to cry; crying lets out emotions, but it doesn’t resolve anything, so I’m trying to be stronger and I’m not at that point yet.” She reported that she was under stress even prior to diagnosis and attributed this to putting the needs of her family before her own and discounting her own needs. She felt that one of her greatest strengths was her ability to achieve positive states of mind, such as peace, which was her helper and aspirational life purpose: “My aspiration was peace…I need to accept the things I can’t change and [have] the courage to change the things I can and have the wisdom to know the difference …” She was open to adopting new perspectives and to restructuring negative thought patterns into patterns more aligned with her aspiration of peace.

#### 3.1.3. Theme-Related Findings

For Jan (LC), there was a connection evident between unexplored emotions and the decrease in her SWB and QL. The basis for this finding is that even during the interview, Jan was flooded with emotions concerning her breast cancer experience. We hypothesized that these emotions had yet to be fully integrated into a cohesive narrative of the cancer experience, which may have negatively impacted both her psychological and spiritual well-being: She continued to struggle emotionally and spiritually with cancer; “[In the past] I turned things over to God, but I haven’t reached that with this [cancer] situation.” Jan stated that PSIT provided an opportunity to reflect on her life and learn new tools for reducing stress. Creative expression (journal writing) facilitated her process of cognitive restructuring. Further supporting this theme was Jan’s use of PSIT techniques including working with her helpers and hinderers, developing witness- non-judgmental acceptance, surrendering, and her aspirational life purpose. Other aspects of mindfulness, such as acknowledgement and letting go, played an important role for her as well in cultivating alternative perspectives.

### 3.2. Beth (LC)

#### 3.2.1. Spirituality

Beth (LC) had a FACIT-Sp-EX pre score of 53 and a post score of 59, which was an 11.32% increase in SWB. Her FACT-B pre score was 98 and her post score was 101, a 3% increase in QL. She described herself as non-religious but believed in the presence of God in her life and had an active yoga and meditation practice. Beth described her meditation practice as “opening myself to allow God to be the center of my life and absorb the love and peace… and to spread love and peace to the world.” She reported that her meditation practice deepened during her cancer experience but that she noticed some resistance to practicing meditation and was easily distracted. She described a belief based on a yogic principle rooted in Raja Yoga meditation that everyone is at peace but that people must continually return to this remembrance because they forget. She also created a life prayer, detailing what she wanted God to bring into her life, which was that she read daily in the morning and before bed.

#### 3.2.2. Psychological Functioning

Beth (LC) was told by a health care provider that stress was the main cause of her cancer; because of this, Beth believed that her high stress levels needed to be addressed. Prior to beginning PSIT, Beth engaged in several healing modalities to improve her psychological functioning, including life coaching, journaling, and psychotherapy. She also participated in other breast cancer support groups prior to PSIT. Per her self-report, she resisted engaging in some of the suggested practices during her participation in all of these other healing modalities.

##### Theme-Related Findings

In her difficult moments, Beth (LC) believed that God would not let her down, and that perhaps God was testing her until she could not take it anymore. One such test was when Beth’s mother was unwell and Beth was unable to care of her because she was overseas. “I can’t take care of her physically [so] it’s comforting- the idea [of God having a reason]…I just think I [am] taking care of her in a way so, that’s comforting, or if I die, then, maybe God says that your role in earth is done.” Beth often inquired about the intentions of God with laughter and genuine puzzlement, “sometimes when I’m facing difficulties…I [think] oh what is God doing here?...why do I have to go through this kind of stuff! (laughs)...why can’t life just be easy? (laughs).”

Additionally, Beth reported that she did not find the mindfulness meditation audio CD that was supplied for home use to be helpful. As mindfulness was a key component of the treatment intervention, this may partially explain her treatment outcome. Because Beth did not perform the mindfulness meditations as outlined in the PSIT intervention, she likely did not receive many of the benefits of mindfulness practice reported in the literature.

### 3.3. Carol (HC)

#### 3.3.1. Spirituality

Carol (HC) had a FACIT-Sp-EX pre score of 40 and a post score of 61, which was a 52.5% increase in SWB. Her FACT-B pre score was 69 and her post score was 99, a 43% increase in QL. She was raised Christian but reported rejecting Christianity after her mother’s death when she was in her early adolescence. Prior to starting PSIT, she reported participating in a life coaching class, a cancer writing group, and a breast cancer book group. Carol had practiced yoga since her 20s and she continued practicing several times a week when she was in treatment for cancer. She described her spirituality as pantheist, nature oriented, ethical, and connected with the world: “I always thought of myself as a Pantheist…nature is the product of the Creator and so in practicing yoga I think you can celebrate the spirituality in that way as part of…the living world.” She connected yoga, meditation, and writing with her spirituality because these activities allowed her to be at one with her creator.

#### 3.3.2. Psychological Functioning

Carol (HC)’s psychological functioning is closely connected with her spiritual practices; she appears to have cultivated positive psychological functioning through these practices. She described an essential part of her spiritual practice as letting go, and stated that through this process she was able to let go of her “to-do list and agenda” and listen to her inner voice. By listening to her inner voice, Carol was able to engage in her creative writing, which brought her a sense of relief. Carol reported that in the past she had struggled with her inner critic, which she labeled as her hinderer. Through PSIT and her spiritual practices, she described being able to develop tolerance and forgiveness for her own imperfections. PSIT also helped her to become more aware of her spirituality in daily life “I think maybe [I became] more aware of the spiritual aspect to what I was doing and [it felt] more integrated into my everyday life.”

#### 3.3.3. Theme-Related Findings

One of Carol (HC)’s unique strengths was her ability to utilize yoga poses for healing. For example, she used the yoga warrior pose as a helper during the PSIT workshop, stating that she was drawn to the warrior’s strength, courage, and determination. She reported that while doing yoga poses she was able to dialogue with both the helping and hindering parts of herself and arrive at new insights. Carol was also able to stop herself from becoming stuck on strong emotions, such as anger, by working through them with creative expression such as poetry in her journal. Carol used writing to elucidate the desires of the helper and the hinderer by writing creatively from their point of view; this process helped her to accept both parts of herself and their unique roles. Through this process, she learned to accept her own imperfections as well as the imperfections of others, leading to forgiveness, “I find that in accepting my own imperfections it is a lot easier to accept other people’s imperfections, it’s a lot easier to forgive other people once you’ve forgiven yourself, which is something I have known since forever, (laughs) but you have to forgive yourself first and then you can forgive the other person.”.

### 3.4. Dee (HC)

#### 3.4.1. Spirituality

Dee (HC) had a FACIT-Sp-EX pre score of 58 and a post score of 87, which was a 50% gain in SWB and the highest post-test score for any of the 4 participants in this study. Her FACT-B pre score was 101 and her post score was 123, a 22% increase in QL. Dee identified as a Christian. She viewed God as a kind, loving father who wanted to help her to have a loving relationship with him. She also viewed God’s role as helping her find direction and purpose in her life.

#### 3.4.2. Psychological Functioning

During the interview, Dee (HC) reported experiencing significant stressors related to family life and physical health through the course of the PSIT workshop. She described the emotional toll that these challenges took on her and how they impacted her PSIT experience. She articulated using the transformative work in PSIT to overcome interpersonal challenges and to establish healthier relationships. She stated that the PSIT experience enabled her to feel more vulnerable, which in turn allowed to her develop a greater capacity to love and be loved by others in her life.

#### 3.4.3. Theme-Related Findings

Dee (HC) believed that her strong religious background gave her a context for understanding the psycho-spiritual experiences that she had in the PSIT intervention and helped her integrate these experiences into her spiritual and psychological framework. “My life has always been kind of spiritual and religious. I think that the thing that [PSIT] has done for me the most is [with] my own stubbornness (laughs)…, sitting back and taking the time for that meditation and acknowledgement of… the gratitude and the recognizing … the little ways that I am blessed.” Dee demonstrated increased levels of psychological and spiritual awareness and insight as well as increased ability to tolerate strong emotions following the intervention. During the interview, she stated that she recognized that she had more ability to recognize self-doubt and to allow these feelings to move through her without overwhelming her.

Dee noted that listening to the needs of her helper and the hinderer allowed her to become more tolerant of the parts of herself that were difficult to accept before. This new acceptance led to a greater ability to manage stress, overcoming multiple personal obstacles, and develop new interpersonal behavior patterns. Further, she demonstrated determination and affect tolerance in persevering through the workshop despite being limited by two physical injuries. She stated “It was very hard for me because physically I ended up being quite sick through most of it, so there were times when it was quite hard to make the effort to come down here and to do it, and yet I thought it was important and I wanted to and I got something out of it, but there were times that the effort to get here was like ah, can I do this and then but I would always go home feeling better.” PSIT core components also helped her grow psychologically and spiritually: “[PSIT] deepened my relationship and my understanding and [it made me] feel more like who I really am and who I am supposed to become.” 

### 3.5. Results of the Multiple Case Analysis

Individual case study findings were merged into multiple-case observations that address the research questions.

#### 3.5.1. Theme 1: Participant Factors

A common finding across cases related to Theme 1 is the role of psychological processes in relation to change in SWB and QL. Participants exhibiting high levels of psychological resistance to elements of the PSIT process were more likely to experience psychological and emotional distress. Such defenses may have reduced openness to PSIT experiential activities that rely heavily on spiritual methods, such as meditation.

The extent to which a participant had a preexisting spiritual framework that influenced meaning-making may have either promoted or inhibited positive or negative SWB and QL change. While each of the participants described having some form of spiritual background, their interpretation of their spiritual system and the extent to which they engaged in certain types of practices may be related to SWB and QL changes.

#### 3.5.2. Theme 2: PSIT Process Components

Participants described a number of components of the PSIT intervention as essential factors in their experiences, including the concepts of the personal helper and hinderer of achieving their aspirational life purpose, witnessing, acknowledgement, integration, letting go, movement, meditation, creative expression, and yoga. The PSIT model gave participants an alternative way of viewing and interpreting their experiences.

#### 3.5.3. Theme 3: Relationship between SWB and QL

In each case, the SWB percentage change had a similar percentage change in the QL score. That is, low changes in SWB −7.5% and +11.5% corresponded to low changes in QL −3% and +3%. Conversely, those with higher changes in SWB +52.5% and +50% had higher changes in QL +43% and +22%. This relationship was also corroborated in the interviews. Engagement with and use of spiritual techniques such as prayer, meditation, yoga, creative expression, and other methods of spiritual practice may be linked to both SWB and QL enhancements.

## 4. Discussion

The findings are interpreted first at the individual case level and then at the multiple case analysis level. We also consider the study’s limitations and recommend directions for future research.

### 4.1. Interpretation of Findings at the Individual Case Level

#### 4.1.1. Jan (LC), Theme 1: Participant Factors

Study findings yielded several participant factors that may partially explain Jan’s low SWB change scores. First, Jan used two prayer practices, ritual prayer and petitionary prayer, which are associated with lower levels of psychological well-being. McCullough and Larson found that those who engaged in frequent ritual prayer tended to have higher levels of negative affect (r = 0.14) and slightly lower levels of well-being [30]. In addition, petitionary prayer, defined as asking God to meet the particular needs of oneself or significant others, has been described as a marker for psychosocial distress when utilized alone to ask God for direct intercession subsequent to a negative life event [31].

Jan’s struggle to overcome rather than accept her emotions and her tendency to judge herself for having strong emotions was a theme that ran through the interview data. In the interview data, Jan stated that she should not be crying, stressed, and worried about her experiences because these emotions did not accomplish anything for her. This belief is contrary to the mindfulness foundation of nonjudgmental acceptance. Jan stated several times during the interview that she recognized a need to accept her breast cancer experience but had difficulty doing so. Additionally, this interview data may indicate that Jan’s non- judgmental witness consciousness was not fully developed, and this might also have interfered with her ability to engage in mindfulness exercises. Finally, Jan’s tendency towards self-judgment may have impeded gains in SWB and QL; previous research has suggested that feelings such as non-forgiveness and resentment are detrimental to physical, mental, and spiritual health [32,33].

#### 4.1.2. Jan (LC), Theme 2: PSIT Factors

Study findings also indicated that some PSIT intervention factors might have contributed to Jan’s low SWB change score. Jan described successfully using cognitive restructuring to contextualize her experience. This may have improved her well-being, since previous research has suggested that cognitive restructuring may enhance the emotional well-being of women with breast cancer [34]. However, while the PSIT intervention may have helped Jan refocus her worry and find short-term solutions, this may have provided only temporary relief from her suffering.

#### 4.1.3. Jan (LC), Theme 3: Relationship between SWB and QL

Jan reported positive changes in her religious and spiritual practices during the interview but her total SWB and QL were not consistent with her reported experience. Her scores declined at each measurement point.

#### 4.1.4. Beth (LC), Theme 1: Participant Factors

Like Jan (LC), Beth (LC) utilized ritual prayer and a pleading approach similar to a petitionary style of prayer, which may have contributed to her lower QL and SWB change scores. Additionally, Beth described a part of her spiritual practice that involved surrendering everything to God. Cole and Pargament described a type of surrender that involves increasing one’s sense of personal control while recognizing limitations; this type of surrender has been tied to an active coping style [35]. However, Beth’s experience of surrender appears to be similar to a process called deferring. According to other researchers, this is a less effective coping style in which the participant relinquishes personal responsibility and turns everything over to God [31]. It is conceivable that this passive coping practice contributed to Beth’s low change scores.

#### 4.1.5. Beth (LC), Theme 2: PSIT Factors

One element of PSIT that was helpful for Beth was learning to view herself as the witness or observer. She reported that this helped her reduce stress perhaps because of her feeling that she needed more focus in her life. Beth also reported that breath awareness helped her calm her physiological response to stress and to redirect her awareness to the present moment. Beth did note, however, that the mindfulness practice in PSIT was not helpful to her. Beth’s difficulty engaging with the mindfulness practices in PSIT may have prevented her from gaining the full stress-reduction benefits and may have contributed to her low SWB and QL change scores when compared to the high change scorers.

#### 4.1.6. Beth (LC), Theme 3: Relationship between SWB and QL

PSIT’s emphasis on the integration of existing spiritual practices into one’s life may have created an opportunity for Beth to utilize these practices for SWB and QL enhancements. Beth’s improvements in these areas provide further support of previous research demonstrating the positive relationship between spirituality, health, and QL [36,37,38,39].

#### 4.1.7. Carol (HC) Theme 1: Participant Factors

Carol (HC) practiced yoga both before and during the PSIT intervention. Culos-Reed *et al*. describe benefits of yoga in healing from breast cancer that include increases in global QL and emotional functioning [40]. Carol also used imagery, poetry, and creative journaling to release emotions. Releasing emotions through engaging in creative activity has been shown to enhance psychological well-being in breast cancer patients [36]. Finally, Carol displayed resilience, affect tolerance, and emotion regulation skills when she faced multiple life stressors during the course of the PSIT workshop. Mindfulness practice has been shown to strengthen and develop these skills, and Carol’s pre-existing skill set may have been amplified by her participation in PSIT [41].

#### 4.1.8. Carol (HC), Theme 2: PSIT Factors

Carol demonstrated an ability to use creative and empathic writing to increase her acceptance of adversity and forgiveness. This finding is consistent with previous literature reporting that the act of writing about trauma can increase physical and mental health [42]. Other literature suggests that acceptance, forgiveness, and empathy improves QL, SWB, and emotional functioning [43]. PSIT also provided Carol with tools she can utilize in the future when coping with adversity or conflict; she noted that these tools taught her to pause and access a deep inner peace from which to respond to adversity. These benefits of utilizing mindful awareness are consistent with previous literature [41].

PSIT’s emphasis on meditation impacted Carol’s spiritual life by promoting “silence”. In the interview she quoted, “Be silent and know that I am God.” PSIT taught her to connect with this silence through the integration of meditation and yoga into her daily life. Carol noted the example of meditating while stopped at a traffic light, stating that this opened a direct line to the spiritual. This method of informal mindfulness practice and its utility has been described by Kabat-Zinn [44].

Carol stated that the PSIT group atmosphere was positive and different from previous experiences she had in meditation groups. She noted that she appreciated the fact that the facilitator allowed participants to do most of their work internally, providing an environment in which members were buffered from other group members’ interpersonal stressors. This finding is consistent with previous research suggesting positive group therapy outcomes are related to group composition and setting [45]. She appeared to have formed a strong therapeutic alliance with the facilitator, an important factor in treatment outcome [46].

#### 4.1.9. Carol (HC), Theme 3: Relationship between SWB and QL

Carol began the study with SWB on the lower end of the spectrum and therefore had potential for a significant increase in SWB. She showed significant improvements in both SWB and QL over the course of the study. Pre-existing spiritual inclinations may have contributed to a high level of interest, curiosity, and dedication to her transformation and the spiritual practices in the workshop.

#### 4.1.10. Dee (HC), Theme 1: Participant Factors

Dee had a strong spiritual background that likely contributed to her ability to benefit from PSIT and to her experiencing increased SWB and QL. Previous findings in the spirituality and health literature have suggested spiritual involvement has a positive association with health and a negative relationship to the severity of psychiatric disorders [39]. Other researchers have described the role of a strong spiritual background promoting SWB and QL [47]. Dee was able to recognize and make light of psychological processes such as self-doubt while they were occurring, and was able to move through them by doing so. She progressed through the workshop despite having two physical injuries, which demonstrated a strong capacity to tolerate and move through challenges. It is likely that Dee was functioning well prior to PSIT participation, as her QL total score was nearly seven points above the combined groups pretest total score mean. This may have enabled Dee to utilize PSIT methods such as mindfulness practices to positively transform SWB and QL.

#### 4.1.11. Dee (HC), Theme 2: PSIT Factors

An important experience from Dee’s perspective was the facilitator mentioning that Dee’s eyes looked bright. She stated that this was a validating experience that helped her feel like she was at a good place in her life. Dee also described the importance of the PSIT loving-kindness practice during the interview. The importance of this experience was demonstrated in how she described receiving the gift of love in a meditation. This finding is consistent with research that indicates that increased mindfulness can lead to purpose in life, social support, and decreased illness symptoms [48,49]. Other researchers have suggested that increases in positive emotions might increase one’s personal resources and thus contribute to increased life satisfaction and reduced depressive symptoms [49].

Dee (HC) described a profound experience working with her witness. In a meditation session, the image of a gift came into her mind offered to her by her experience of herself as a witness. She has come to understand the gift as a message that she should love herself so that she can love “her father in heaven” and others, and so that she can reach out to others with love. This illustrates the mechanism of the witness and compassion within the PSIT model [22]. In PSIT, participants work with the witness to observe various parts of the self, including the helper and the hinderer. By embodying the witness in this manner, perhaps Dee was able to cultivate this mental image and create a psychological state of openness, which allowed this gift to emerge.

#### 4.1.12. Dee (HC), Theme 3: Relationship between SWB and QL

Based on Dee’s data, there may be a connection between her SWB changes and her QL changes; as she developed through spiritual work, she grew in her self-efficacy of feeling capable of being able to love and be loved. For Dee, this change brought about more positive feelings and an increased realization of the need to take better care of herself. For her, positive self-care included improving her relationship boundaries to increase the positivity of those relationships. Mindfulness is shown to improve relationship satisfaction, perceived closeness, and acceptance of oneself and one’s partner [50]. Additionally, Dee reported that the technique of breath-awareness was helpful in facilitating stress reduction and present-moment centeredness. Dee partially attributed her increased ability to control her blood pressure to the breathing practices she learned in PSIT.

### 4.2. Multiple Case Study Assertions

The multiple case analysis yields assertions that synthesize individual case findings and themes (participant and PSIT intervention factors) to address the primary aims of the study. Each research finding was examined for each theme and then merged together based upon its meaning and content to formulate a multiple case assertion. These assertions are essentially hypotheses drawn from the results to be tested in future research.

#### 4.2.1. Theme 1: Participant Factors

There are two primary multiple case assertions related to participant factors. The first assertion is that individual participants’ psychological skills contribute to improvements in SWB and QL. Among these skills, one of the most important is one’s ability to acknowledge, tolerate and accept intense emotions. This assertion is aligned with both the literature describing the foundations of mindfulness practice (as taught by Kabat-Zinn) and PSIT, and the PSIT theoretical model [19,22,44]. The second assertion is that participants’ spiritual and religious frameworks contribute to SWB and QL changes. Participants who had an active spiritual or religious practice prior to the intervention, and/or had a strong grasp of spiritual practices, reported greater improvements in SWB and QL. However, spiritual doubts and/or trials were associated with negligible improvement.

#### 4.2.2. Theme 2: PSIT Factors

We developed four assertions related to PSIT intervention factors. The first assertion is that the core components of the PSIT model promote improvements in SWB and QL. Each participant described core components of PSIT such as witnessing, aspiration, life purpose, and helper/hinder as being meaningful in their experience of the intervention. The second assertion is that developing alternate perspectives and practicing cognitive restructuring are beneficial in influencing SWB and QL changes. Several of the participants noted that developing different ways of perceiving their experience was helpful.

The third assertion addressed the positive group model of PSIT as described by the participants. The majority of participant processing occurred internally, freeing the participants from bearing the stress of detailed information disclosed regarding other participants’ experiences of challenging life experiences. The fourth assertion involves the importance of the group facilitator. The group facilitator provides meaningful insight, reflection, and validation for participants both during group sessions and during individual interactions outside of the treatment frame, such as before or after meetings or hallway run-ins. Together, these four assertions highlight the role of PSIT factors in promoting SWB and QL.

#### 4.2.3. Theme 3: Relationship between SWB and QL

The participants who reported the largest changes in SWB (the high changers) also reported the greatest changes in QL, and those who reported the lowest changes in SWB (the low changers) also reported the lowest changes in QL. The main assertion that is drawn regarding the relationship between changes in SWB and QL is that participant use of spiritual techniques influences changes in both SWB and QL. SWB can be enhanced by spiritual practice; however, the connection between spiritual practice and QL is more difficult to determine. Many of the spiritual practices utilized, such as mindfulness meditation and yoga, impact the body’s physiology in a positive way over time. Such positive impact may transfer over to QL enhancements, as QL is linked to physical well-being.

Although participant practice time was not directly measured, the interview data did capture, less objectively, participants’ dedication toward spiritual practice. While each of the 4 participants reported engaging in spiritual practice, the type of spiritual practice seemed to be linked to the outcomes. Specifically, both Jan (LC) and Beth (LC) reported utilizing types of prayer and coping strategies that are associated with less positive outcomes following a life stressor. While this is a complex issue in need of further research, this is consistent with prior literature suggesting that some types of prayer can have negative impact on SWB and QL [31].

### 4.3. Limitations

Multiple case analysis designs call for a small number of participants due to the enormous amount of data that must be analyzed for each participant. To minimize the impact of small sample size, this research must be carefully interpreted within its context: spirituality and spiritual experiences related to a CAM-spiritually integrated psychotherapy intervention for women coping with breast cancer. The generalizability of this study also may be limited by participant demographics; women who chose to participate in this spiritually integrated psycho-social intervention may already be more spiritually oriented and more likely to benefit from PSIT than others.

Additionally, all four participants in this study were previously interviewed at varying time intervals by other PSIT researchers for other studies. The extent to which participants’ descriptions of their PSIT experience were impacted by previous interviews is unknown. Finally, five other participants with lower spiritual well-being change scores either declined participation or were unavailable for study inclusion, leaving open the possibility of undiscovered findings for those with low SWB change scores.

Another factor that is difficult to measure but that may have had influence across cases is the role of the therapist. For example, the therapist may have conversations with individual participants in the hallway during a workshop break that could also contribute to changes in SWB and QL.

It is beyond the scope of this study to fully measure the significance of factors such as group sharing and group dynamics; many participants reflected positively on the group experience and their outcomes may have been influenced by this positive experience. The PSIT omnibus study partially addressed this issue by comparing PSIT to a community based breast cancer group, and future research should include a comparison group intervention that controls for time and attention, preferably with randomization to treatment condition [24].

### 4.4. Recommendations for Future Research

This case study research yields recommendations for future research, overlapping with those of investigators completing research on the PSIT intervention groups [20,23,24]. This study shows the value of using a multiple case analysis that can suggest additional hypotheses about individual and intervention characteristics that can be tested in subsequent research. The results of this multiple case analysis suggests that future studies of PSIT and other psycho-spiritual interventions for women with breast cancer may benefit by including a standardized assessment of spiritual and religious history and involvement, perceived relationship with higher power (where applicable), life meaning and purpose, and spiritual/religious beliefs, motivation, practices, and experiences. Such standardization would facilitate the comparison of findings across studies.

Research on the implementation of PSIT would benefit from the use of multiple facilitators, controlling for treatment adherence, a randomized control design, multiple study locations, and a larger, more representative sample. It is also recommended that future multiple case study analysis research utilize a research team model from the onset of designing the study to provide multiple perspectives on data analysis and conclusions as well as allow for increased sample size.

## 5. Conclusions

These findings suggest that there is a relationship between changes in SWB and changes in QL, with the women experiencing the greatest changes in SWB also showing the greatest improvements in QL. Furthermore, participant changes in SWB and QL in the context of the PSIT intervention appear to be related to participant characteristics including spiritual and religious backgrounds, doubts, and trials as well as the application of metacognitive psychological skills.

Other benefits of PSIT stemmed from use of core components of the intervention. Participants reported they benefited by clarifying an aspirational life purpose and by developing mindful non-judgmental witnessing of the personal factors that helped and hindered the actualization of that life purpose. These processes lead to cognitive restructuring and developing alternative perspectives. In a group setting that emphasized inner processing, participants reported learning a set of skills useful in dealing with cancer survivorship and other life stressors.
